# Nasopharyngeal carriage and antimicrobial susceptibility profiles of *Streptococcus pneumoniae* among children with pneumonia and healthy children in Padang, Indonesia

**DOI:** 10.1099/acmi.0.000584.v3

**Published:** 2023-06-16

**Authors:** Finny Fitry Yani, Riris Juita Julianty, Wisnu Tafroji, Linosefa Linosefa, Indra Ihsan, Nice Rachmawati Masnadi, Dodi Safari

**Affiliations:** ^1^​ Departement of Child Health, Faculty of Medicine, Universitas Andalas, Padang, Indonesia; ^2^​ Departement of Paediatric, Dr. M. Djamil Hospital Padang, Kota Padang, Indonesia; ^3^​ Eijkman Research Center for Molecular Biology, National Research and Innovation Agency (BRIN), Cibinong, West Java, Indonesia; ^4^​ Departement of Microbiology, Faculty of Medicine, Universitas Andalas, Padang, Indonesia

**Keywords:** children, community-acquired pneumonia, nasopharyngeal carriage, streptococcus pneumoniae

## Abstract

*
Streptococcus pneumoniae* is one of the pathogenic bacteria causing invasive pneumococcal diseases such as pneumonia, sepsis, and meningitis, which are commonly reported in children and adults. In this study, we investigated the nasopharyngeal carriage rates, serotype distribution, and antimicrobial susceptibility profiles of *S. pneumoniae* among children with pneumonia and healthy children under 5 years old in Padang, West Sumatra, Indonesia. Nasopharyngeal swabs were collected from 65 hospitalized children with pneumonia in a referral hospital and from 65 healthy children at two day-care centers from 2018 to 2019. *S. pneumoniae* was identified by conventional and molecular methods. Antibiotic susceptibility was performed with the disc diffusion method. Out of 130 children, *S. pneumoniae* strains were carried by 53% and 9.2 % in healthy children (35/65) and children with pneumonia (6/65), respectively. Serotype 19F was the most common serotype among the isolated strains (21%) followed by 6C (10%), 14, 34 (7 % each), and 1, 23F, 6A, 6B (5 % each). Moreover, 55 % of the strains (23/42) were covered by the 13-valent pneumococcal conjugate vaccine. Most isolates were susceptible to vancomycin (100%), chloramphenicol (93%), clindamycin (76%), erythromycin (71%), and tetracycline (69%). Serotype 19F was commonly found as a multi-drug resistant strain.

## Data Summary

Supplementary materials accompany this paper: File S1: Data Serotype and Antimicrobial Susceptibility Test.

## Introduction

Pneumonia, an infection of the lung parenchyma, still remains a major cause of morbidity and mortality in children worldwide [1]. Pneumonia is the leading cause of death for children under 5 years of age that killed 740 180 children (14 % of all deaths of children under 5 years of ages) worldwide in 2019, occurring mainly in developing countries [2]. Pneumonia also results in one of the largest state expenditures both directly, through medical costs and indirectly, by the loss of working hours in the parents taking care of their sick children [3]. In Indonesia, 16 % of children's deaths (aged between 29 days to 11 months) in 2019 were due to pneumonia followed by diarrhoea [4]. Meanwhile, pneumonia (9.5 %) is the second largest cause of death in children between aged 12 to 60 months under five [4]. *Streptococcus pneumoniae *, as one of the causative agents of pneumonia, is an opportunistic pathogen colonizing the human nasopharynx [5]. This colonization can increase risk of infection depending on the immune system’s condition [5, 6]. In this study, we investigated the nasopharyngeal carriage rates, serotype distribution, and antimicrobial susceptibility profiles of *S. pneumoniae* among children with pneumonia and healthy children under 5 years of age in Padang, West Sumatra, Indonesia.

## Methods

### Study design and specimen collection

The study was conducted at the Dr. M. Djamil Hospital, a provincial referral hospital, and at two day-care centres located in Padang, West Sumatra, Indonesia. The children enrolled in the Dr. M. Djamil Hospital were admitted patients with clinical pneumonia from April 2018 to December 2019. The enrollment criteria of children with pneumonia included new or progressive infiltrates on chest radiographs with ≥2 of the following criteria: dyspnoea, cough, hemoptysis, chest pain, and fever occurring ≤14 days before admission. Healthy children under 5 years of age, who were attending day-care centres, were also enrolled from August to December 2019. Demographic characteristics and clinical data were collected and recorded in the case report form.

The Nasopharyngeal (NP) swab specimens were collected from both groups using flocked nylon swabs (Copan; Cat. No. 503CS01) placed into 1 ml skim milk-tryptone-glucose-glycerol (STGG) medium followed by vortex. Samples were then stored at −80 °C. The specimens were regularly shipped to the Eijkman Centre for Molecular Biology (Eijkman Research Centre for Molecular Biology), Jakarta with dry ice for the isolation and identification of *S. pneumoniae *.

### Bacterial identification

The NP swab specimens were enriched by transferring 200 µl of swab-STGG medium into 6 ml enrichment media consisting of 5 ml Todd-Hewitt broth (BD Bacto; Cat. No. 249240) with 0.5 % yeast extract (BD Bacto; Cat. No. 212750) and 1 ml rabbit serum (Gibco; Cat. No. 16120099), and then were incubated at 37 °C with 5 % CO_2_ for 5 h. A 10 µl of the enriched specimens was inoculated onto a sheep blood (8%) agar plate (sBAP, comprised of TSA II; BD cat. no. 212 305, with 8 % v/v sheep blood supplementation) and incubated at 37 °C with 5 % CO_2_ for 20 h [7]. After that, all blood agar plates were examined for suspected *S. pneumoniae* colonies with the following colony morphologies: alpha-hemolysis, mucoid, and depressed centre. Suspected colonies were streaked on a sBAP and a disc containing 5 µg of optochin was placed onto the inoculated media. Colonies susceptible to optochin (inhibition zone diameter >14 mm) and positive for bile solubility were identified as *S. pneumoniae *.

Bacterial DNA was extracted by enzymatic fast DNA extraction as follows: the overnight colony on BAP was resuspended in 300 µl of NaCl 0.85 % and the suspension was vortexed. This mixture was then incubated at 70 °C for 15 min followed by centrifugation at 10 000 r.p.m. for 2 min. The supernatant was discarded and the pellet was then resuspended with 50 µl Tris-EDTA (TE) buffer followed by homogenization. A volume of 8 µl hyaluronidase (30 mg ml^−1^) and 12 µl mutanolysin (2500 U ml^−1^) were added and the suspension was vortexed. The suspension was then incubated for 30 min at 37 °C followed by enzyme inactivation at 100 °C for 10 min. The mixture was then centrifuged at 10 000 r.p.m. for 4 min and the supernatant containing the DNA was stored at −20 °C until further analysis. Serotype determination was performed by a sequential multiplex PCR (SM-PCR) as published by Carvalho *et al*. followed by Quellung reaction method as described previously [5]. The serogroup six results obtained by PCR was further tested by using enzymatic restriction digest to distinguish serotypes 6A, 6B, 6C, and 6D [8].

### Antimicrobial susceptibility testing

All *S. pneumoniae* strains isolated from both groups were tested for antimicrobial susceptibility using the disc diffusion method according to the Clinical and Laboratory Standard Institute, 2019 [9]. In this study, seven antimicrobial discs (Oxoid) were used: erythromycin (15 µg), sulfamethoxazole-trimethoprim [co-trimoxazole] (1.25/23.75 µg), clindamycin (20 µg), chloramphenicol (30 µg), tetracycline, (30 µg), vancomycin (1 µg), and oxacillin (1 µg).

## Results

The NP swab specimens were collected from a total of 130 children; 65 hospitalized children with pneumonia (mean age of 11.2±10.5 months) during the one and half years period of study, and 65 healthy children (mean age of 29.4±16.1 months) that were recruited from daycares for a period of 4 months. In this study, the proportion of infants less than 1 year of age was 80.0 % (52/65) for the admitted patients and only 13.8 % (9/65) for healthy children. The children’s characteristics from both groups are shown in Table 1. The majority of the hospitalized children’s clinical symptoms were fever (72.3 %), rhonchi (76.9 %), and cough (66.2 %). Most of the hospitalized children had history of previous antibiotics use (54/65; 83.1 %) and chest X-ray infiltrates (56/65, 86.2 %) (Table 1). The good nutritional status of healthy children was higher than children with pneumonia (72.3 % vs 53.8 % respectively).

**Table 1.** Baseline characteristics and bacterial *Streptococcus pneumoniae* findings among children with pneumonia and healthy children in Padang, West Sumatra, Indonesia

Variables		Children with pneumonia (*N*=65) n (%)	Healthy children (*N*=65), n (%)
Basic demographics			
Sex			
	Male	40 (61.5)	36 (55.4)
	Female	25 (38.5)	29 (44.6)
Age (month)			
	0–12	52 (80.0)	9 (13.8)
	13–24	5 (7.7)	24 (36.9)
	25–60	8 (12.3)	32 (49.2)
Exclusive breastfeeding			
	Yes	39 (60.0)	49 (75.4)
	No	22 (33.8)	16 (24.6)
Nutritional status			
	Malnutrition	29 (44,6)	18 (27.7)
	Good	35 (53.8)	47 (72.3)
Number of family member			
	1–3	No Data (nd)	33 (50.8)
	4–6	nd	30 (46.2)
	>7	nd	2 (3.1)
Smoking exposure			
	Yes	nd	45 (69.2)
	No	nd	20 (30.8)
Clinical symptom			
	Cough	43 (66.2)	nd
	Fever	47 (72.3)	nd
	Rhonchi	50 (76.9)	nd
	Chest X-ray infiltrates	56 (86.2)	nd
History of antibiotics			
	Yes	54 (83.1)	nd
	No	11 (16.9)	nd
Bacterial carriage in the nasopharynx			
Positive *S. pneumoniae *		6 (9.2)	35 (53.8)

ND, No Data.

**Fig. 1.**
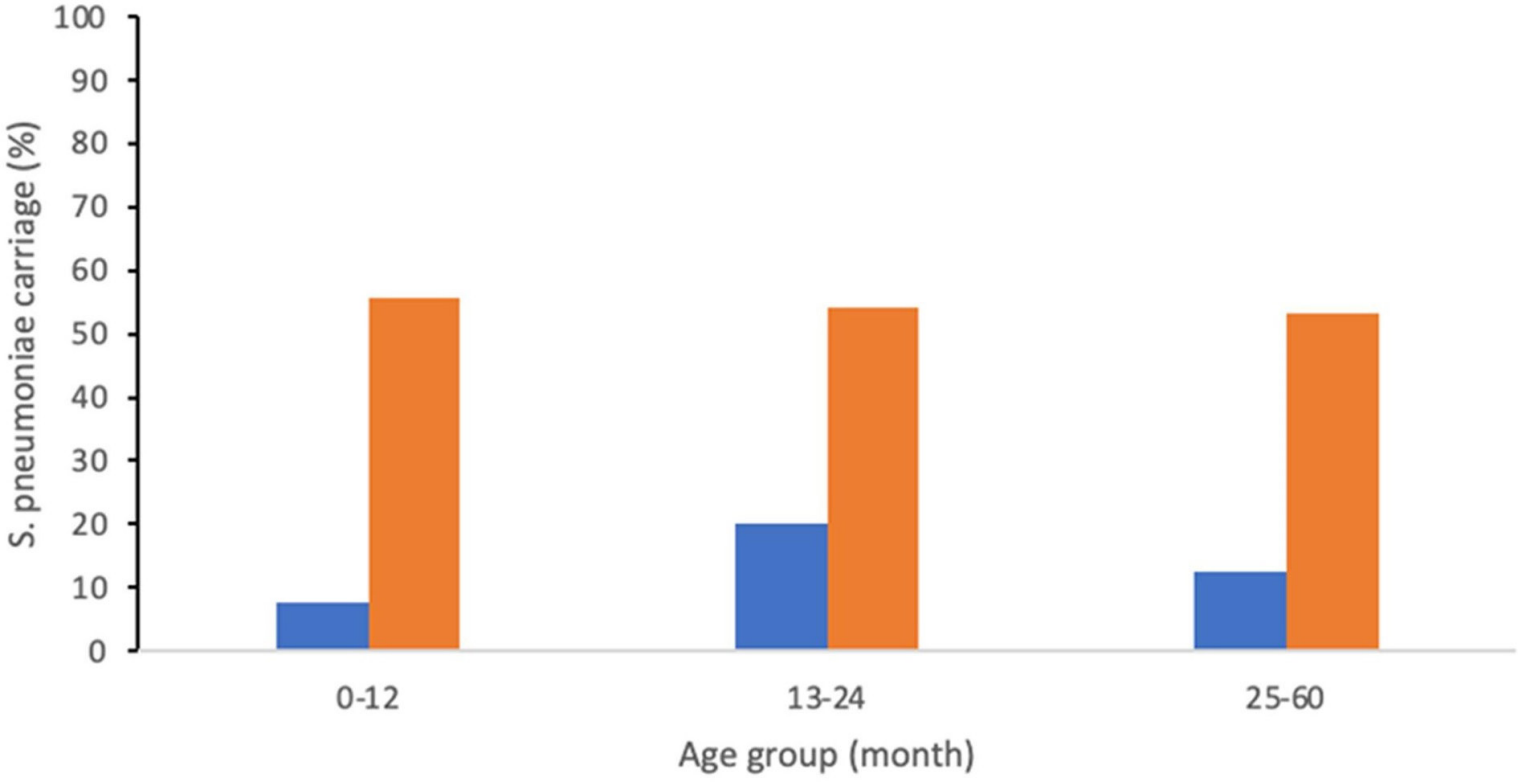
Prevalence of *S. pneumoniae* carried by children with pneumonia (blue bar) and healthy children (orange bar) based on age groups: 0–12 months, 13–24 months, and 25–60 months.

In this study, we found that the nasopharyngeal carriage of *S. pneumoniae* was 9.2 % (6/65) among the children with pneumonia and 53 % (35/65) among the healthy children enrolled from the day-care centres. Among children with pneumonia, the *S. pneumoniae* carriage was different between age groups (Fig. 1). The carriage rates were 7.7, 20.0, and 12.5 % for the age groups of less than 1 year of age (4/52), 13 to 24 months (1/5), and 25 to 60 months (1/8), respectively. Meanwhile, among healthy children, the *S. pneumoniae* carriage rates were 55.6, 54.2, and 53.1 % for the age groups of less than 1 year of age (5/9), 13 to 24 months (13/24), and 25 to 60 months (17/32), respectively (Fig. 1).

**Fig. 2.**
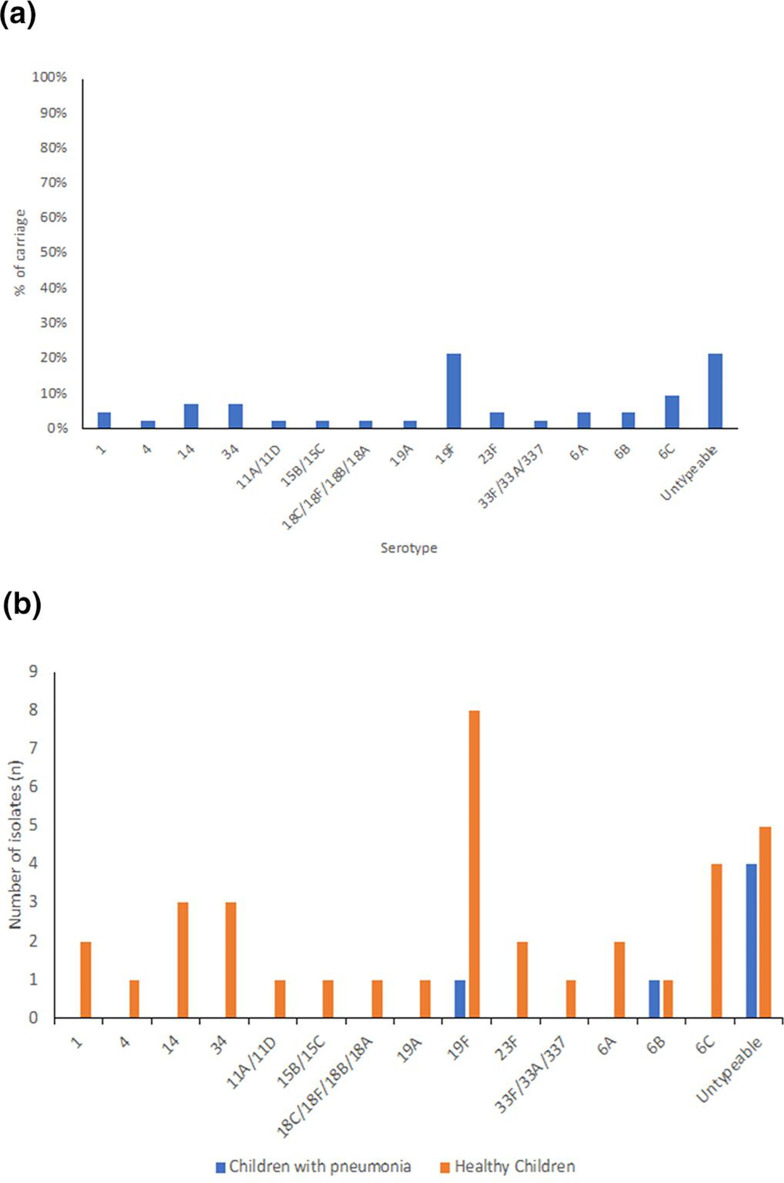
Serotype distribution. (**a**) Among 42 *S. pneumoniae* carriage isolates of children with pneumonia and healthy children in Padang, West Sumatra, Indonesia, (**b**) serotype distribution of each group.

A total of 42 *S. pneumoniae* strains were isolated from 41 positive NP swab specimens, with a single sample (PDG 98) from one healthy-children simultaneously positive for strains of serotype 14 and 19A (Table S1). Serotype 19F was the most common serotype among the cultured strains (21 %; 9/42) followed by 6C (10 %; 4/42), 14, 34 (three carriers each; 7 % each), 1, 23F, 6A, 6B (two carriers each; 5 %), and 4, 11A/11D, 15B/15C, Serogroup 18, 19A, 33F/33A/37 (one carrier each, 2 %) (Fig. 2a). We found that nine isolates (21 %) were untypeable (UNT) strains using the SM-PCR method. Among children with pneumonia, we isolated six *S. pneumoniae* isolates including serotype 19F and 6B (one strain each) and four UNT strains, while among healthy children, we identified thirty-six *S. pneumoniae* isolates with serotype 19F as the most prevalent serotype among cultured strains (8/36) (Fig. 2b). In this study, *S. pneumoniae* strains that were covered by the pneumococcal conjugate vaccine (PCV13) was 50 %.

**Fig. 3.**
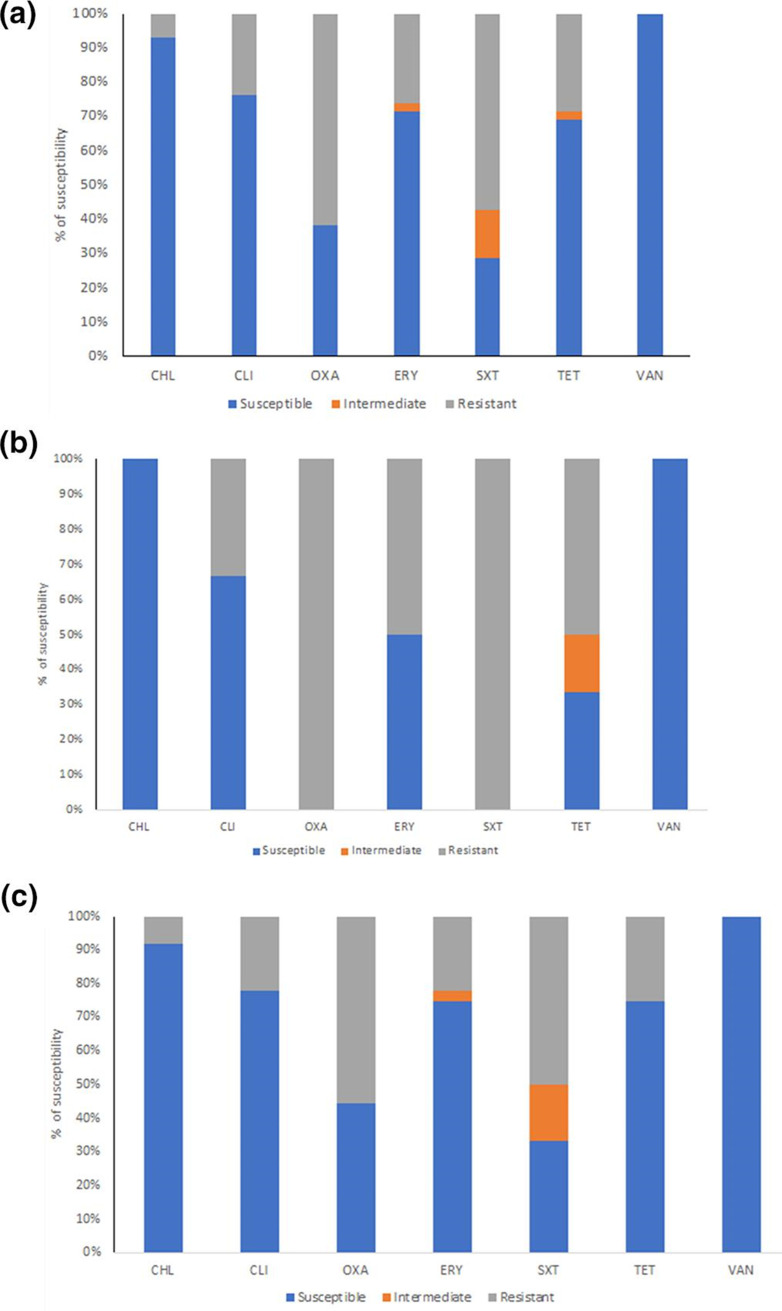
Antimicrobial susceptibility profile of *S. pneumoniae* isolates in Padang, West Sumatra, Indonesia. (**a**). Antimicrobial susceptibility profile of total *S. pneumoniae* strains found in this study. (b) Antimicrobial susceptibility profile of *S. pneumoniae* strains isolated from children with pneumonia. (**c**). Antimicrobial susceptibility profile of *S. pneumoniae* strains isolated from healthy children. CHL: Chloramphenicol; CLI: Clindamycin; OXA: Oxacillin; ERY: Erythromycin; SXT: Trimethoprim/Sulfamethoxazole; TET: Tetracycline; and VAN: Vancomycin.

We found that the majority of *S. pneumoniae* isolates were susceptible to vancomycin (100 %), chloramphenicol (92.9 %), clindamycin (76.2 %), erythromycin (71.4 %), tetracycline (69 %), oxacillin (38.1 %) and co-trimoxazole (28.6%) (Fig. 3a). Among *S. pneumoniae* isolated from children with pneumonia, we found that all isolates were resistant to oxacillin and co-trimoxazole (100 %) followed by tetracycline (67 %), erythromycin (50 %) and clindamycin (33 %) (Fig. 3b). Concordance with *S. pneumoniae* isolated from healthy children, we also found that the isolates were resistant to oxacillin and co-trimoxazole followed by tetracycline, erythromycin and clindamycin (Fig. 3c). Vancomycin is found susceptible for all of isolates from healthy children and children with pneumonia (Fig. 3b and Fig. 3c).

**Fig. 4.**
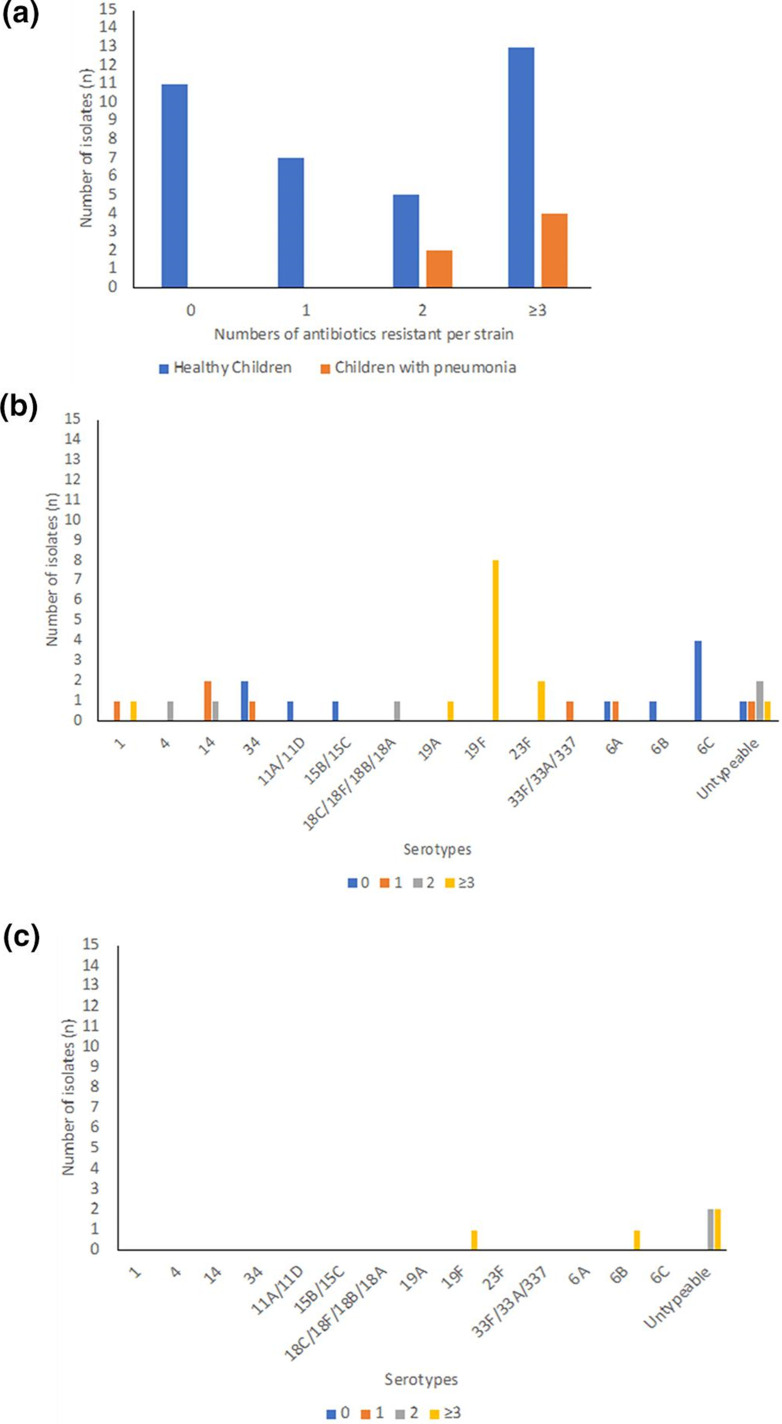
The antimicrobial resistance of *S. pneumoniae* isolates. (**a**) Number of isolates with resistance number, (**b**) numbers of resistance serotypes of isolates from healthy children, (**c**) numbers of resistance serotypes of isolates from children with pneumonia.

Furthermore, we found that there were 36 % (13/36) and 67 % (4/6) of *S. pneumoniae* grouped as multi-drug resistant (MDR) with resistance to ≥3 groups of antibiotics among isolates of *S. pneumoniae* isolated from healthy children and children with pneumonia respectively (Fig. 4a). The serotype 19F is the most common serotype found as MDR (Fig. 4b, c).

## Discussion

In this study, we found that the prevalence of *S. pneumoniae* in children with pneumonia (9.2 %) was lower than healthy children (53 %) in Padang, West Sumatra, Indonesia. A possible explanation for this was due to previous antibiotic use at the secondary level hospital before being referred to our third level hospital and majority of children with pneumonia symptoms are under 1 year old (80 %). A previous study in Thailand also reported that children with pneumonia showed lower prevalence of *S. pneumoniae* colonization (54.5 %) compared to community controls (62.5 %) [10]. However, this is in contrast with a previous study in India reporting that the prevalence of *S. pneumoniae* carriage among children with clinical pneumonia was higher (74.7 %) than community children (54.5 %) [11]. In comparison with a previous study, the range of prevalence of pneumococcal carriage among children under 5 years of age in Indonesia was 13.9–68 % [12]. In addition, a previous study reported that attendance of children in daycare showed significant impact on *S. pneumoniae* colonization [13]. Another study reported that incidence of pneumonia was higher in malnourished children and is significantly different in children with no malnutrition as previously reported [14]. Furthermore, malnutrition was reported to correlate with increase severity and fatality of pneumonia case incidence [1].

In this study, we discovered that the serotypes circulating in West Sumatra, Padang are dominated by the invasive serotypes currently covered in the pneumococcal vaccine. More than half of isolates are included in the pneumococcal vaccine conjugate. This is also in concordance with previous studies which reported the common serotypes of *S. pneumoniae* circulating in Indonesia are the vaccine serotypes [15–17]. The vaccine serotypes are common serotypes distributed in Indonesia because Indonesia has not included the pneumococcal vaccine as part of the national routine vaccination programme. Among the vaccine serotypes, this study found that serotype 19F is the most frequent serotype identified. The frequency of each serotype colonizing children's nasopharynx is found to vary across regions in Indonesia. In Kotabaru, Kalimantan and Lombok, West Nusa Tenggara, the most common serotype is 6A/6B [15, 17]. Meanwhile in Bandung, West Java, the 15B/15C is the most common serotype found in healthy children's nasopharynx.

This study also found that most of the isolates are resistant to co-trimoxazole and oxacillin which is also in concordance with recent publications reporting most of the *S. pneumoniae* isolates as being resistant to co-trimoxazole [17–19]. Moreover, we discovered that not only is serotype 19F the most common serotype but is also resistant to more than three groups of antibiotics, identifying serotype 19F as MDR. This serotype has also been reported as the most common serotype with MDR characteristics in recent studies [17, 20].

The limitations of this study are related to the status of the hospital as the tertiary hospital and a referral centre, in which the history of antibiotic administration could not be provided for this study. In addition, in this study, the hospitalized children with pneumonia group was mostly children under 1 year of age, while in the healthy group there were more children over 1 year of age. This might affect the results of the carriage rates of *S. pneumoniae.* Children under 1 year of age are not common in day-care centres compared to children over 1 year of age, causing the healthy group to have varying results of colonization due to the high risk of pneumococcal transmission. In conclusion, prevalence of pneumococcal carriage in hospitalized children with pneumonia is lower than healthy children under 5 years old in Padang, Indonesia and serotype 19F was commonly found as a multi-drug resistant strain.

## Supplementary Data

Supplementary material 1
